# Synergistic blocking of RAS downstream signaling and epigenetic pathway in *KRAS* mutant pancreatic cancer

**DOI:** 10.18632/aging.204031

**Published:** 2022-04-25

**Authors:** Xiaofei Zhang, Tiebo Mao, Haiyan Xu, Shumin Li, Ming Yue, Jingyu Ma, Jiayu Yao, Yongchao Wang, Xiao Zhang, Weiyu Ge, Yanling Wang, Daiyuan Shentu, Liwei Wang

**Affiliations:** 1 State Key Laboratory of Oncogenes and Related Genes, Shanghai Cancer Institute, Department of Oncology, Renji Hospital, School of Medicine, Shanghai Jiao Tong University, Shanghai, China

**Keywords:** pancreatic ductal adenocarcinoma, MEK inhibitor, BET inhibitor, synergistic effect, autophagy

## Abstract

Background: Pancreatic ductal adenocarcinoma (PDAC) is a highly fatal malignancy and lacks effective therapeutic targets. Trametinib is considered to be a promising potential indirectly targeted KRAS inhibitor in PDAC. However, the clinical outcomes were poor. JQ1 displayed a significant synergistic effect when combined with chemotherapy or potential targeted therapy in pancreatic cancer. The impact of Trametinib and JQ1 combination treatment in PDAC remains to be fully elucidated.

Methods: The efficacy of trametinib and JQ1 on cell proliferation and cytotoxicity was assayed in 7 *KRAS* mutant pancreatic cancer cell lines. The cytotoxic effects of drugs either alone or in combination were evaluated using a luminescent cell viability assay. Immunoblot analysis was carried out to investigate changes in p62 and autophagy.

Results: We found that either trametinib or JQ1 alone inhibited the proliferation of some pancreatic cancer cell lines with *KRAS* alterations, irrespective of the mutational loci of *KRAS* and the aberrant status of the other driver genes. The synergistic effects of combination treatment of trametinib and JQ1 were observed in both trametinib-resistant and trametinib-sensitive cells. In trametinib-sensitive PDAC cells, the combined treatment definitely inhibited p62 expression compared with trametinib alone, while LC3 expression at high levels changed little. In trametinib-resistant PDAC cells, the combination of MEK/BET inhibitor dramatically decreased p62 expression compared with single agent, while p62 expression increased after anti-autophagic therapy was added.

Conclusions: Blocking RAS downstream signaling and epigenetic pathway synergistically increases the antiproliferative activity in *KRAS* mutant PDAC cells. Combination therapeutic synergism may induce different cell death modes in different pancreatic cancer subtypes.

## INTRODUCTION

Pancreatic ductal adenocarcinoma (PDAC) is a highly fatal malignancy with a rapid incidence rate worldwide [1]. Approximately 80–85% of PDAC patients have unresectable or metastatic disease at the time of diagnosis [2]. In addition, the genetic and heterogeneity of PDAC make for a lack of effective therapeutic options, leading to a 5-year survival rate of less than 10% worldwide [3]. It is estimated that pancreatic cancer will become the second leading cause of cancer death by 2030 [4].

Studies have revealed that up to 90% of PDAC patients harbor oncogene *KRAS* activating alterations, which play an essential role in PDAC initiation and maintenance [5]. Directly inhibiting *KRAS* seems to be a desirable approach for specifically treating PDAC patients with *KRAS* mutations. However, with the exception of *KRAS* p.G12C specific inhibitors (a mutation merely accounts for 1% of PDAC patients), various attempts to directly inhibit *KRAS* have been unsuccessful [6]. As an alternative approach, targeting KRAS downstream effectors has been clinically explored [7]. Trametinib, as a highly selective MEK1/2 inhibitor, targets mitogen-activated protein kinase (MAPK) signaling which is a main pathway downstream of KRAS; however, a clinical study has been less encouraging when combined with chemotherapy in PDAC patients [8]. The failure of trametinib in PDAC is probably due to the activation of adaptive signaling, resulting in acquired drug resistance. However, whether there are potential epigenetic-based mechanisms regulating drug sensitivity remains to be fully elucidated.

JQ1, an epigenetic reader protein BET inhibitor of bromodomain-containing protein 4 (BRD4) has emerged as a potential modulation agent [9]. In pancreatic cancer, JQ1 has been reported to exert a synergistic effect and induce tumor regression when combined with gemcitabine, HDAC inhibitors, or even PARP inhibitors [10, 11]. Combination therapy based on BET inhibitors is considered to have promising therapeutic potential for pancreatic cancer [12].

In this study, we aimed to explore the impact of trametinib and/or JQ1 on *KRAS* mutant pancreatic cancer and address the potential mechanism.

## RESULTS

### MEK inhibitor trametinib suppresses pancreatic cancer cells

**Figure 1:**
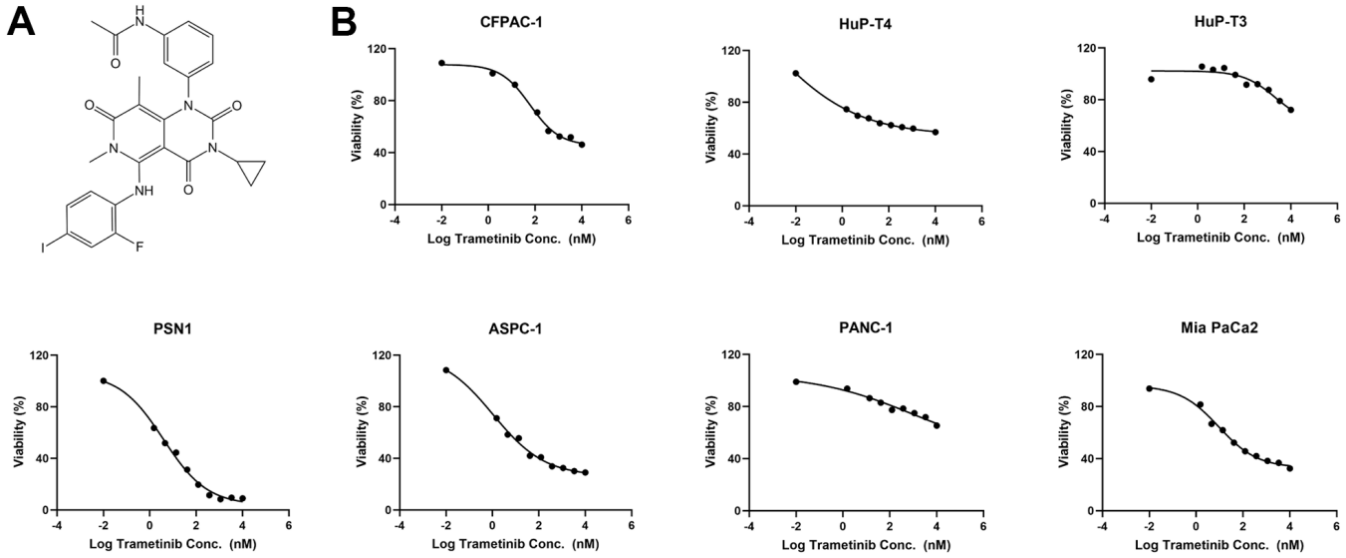
**The MEK inhibitor trametinib suppresses *KRAS* mutant pancreatic cancer cells.** (**A**) The structure of the MEK inhibitor trametinib. (**B**) Fitting curve of cytostatic responses illustrated a decreasing concentration gradient of trametinib in 7 *KRAS* mutant pancreatic cancer cell lines (AsPC-1, MIA PaCa-2, PANC-1, HuP-T4, HuP-T3, PSN1, and CFPAC-1).

First, we demonstrated the structure of trametinib (Figure 1A) and the main genetic alterations in our human PDAC cell lines (Table 1). We found that all 7-cell lines carried *KRAS* and *TP53* mutations. AsPC-1 also had *SMAD4* and *CDKN2A* alterations. PSN1 and CFPAC-1 had *SMAD4* copy number variation (CNV) loss alterations, while Mia PaCa-2, PANC-1, HuP-T3, and HuP-T4 carried *CDKN2A* CNV loss alterations. Then we treated all PDAC cell lines with a decreasing concentration gradient of trametinib. Cytostatic responses were observed in all PDAC cell lines, but the effectiveness was totally different in different cell lines from the fitting curve (Figure 1B). AsPC-1, PSN1, and Mia PaCa-2 cells were relatively sensitive to trametinib, and their half maximal inhibitory (IC50) values were 1.046 nM, 3.866 nM, and 9.167 nM, respectively (Supplementary Table 1). The IC50 values of CFPAC-1 and PANC-1 was 61.22 nM and 1031 nM, respectively, which were relatively resistant to trametinib. However, the IC50 values of HuP-T3 and HuP-T4 were not reached when treated with the maximum concentration of 10 μM trametinib.

**Table 1:** Main mutation analysis of the PDAC cell lines.

**Cell lines**	**KRAS**	**TP53**	**SMAD4**	**CDKN2A**
AsPC-1	p.G12D	p.C135fs	p.R100T	p.L78fs
MIA PaCa-2	p.G12C	p.R248W	wild type	CNV Loss
PANC-1	p.G12D	p.R273H	wild type	CNV Loss
HuP-T4	p.G12V	p.I255T	wild type	CNV Loss
HuP-T3	p.G12R	p.R282W	wild type	CNV Loss
PSN1	p.G12R	p.K132Q	CNV Loss	wild type
CFPAC-1	p.G12V	p.C242R	CNV Loss	wild type

Abbreviations: p, protein; fs, frame shift; CNV, copy number variations.

### BET inhibitor JQ1 suppresses pancreatic cancer cells

**Figure 2:**
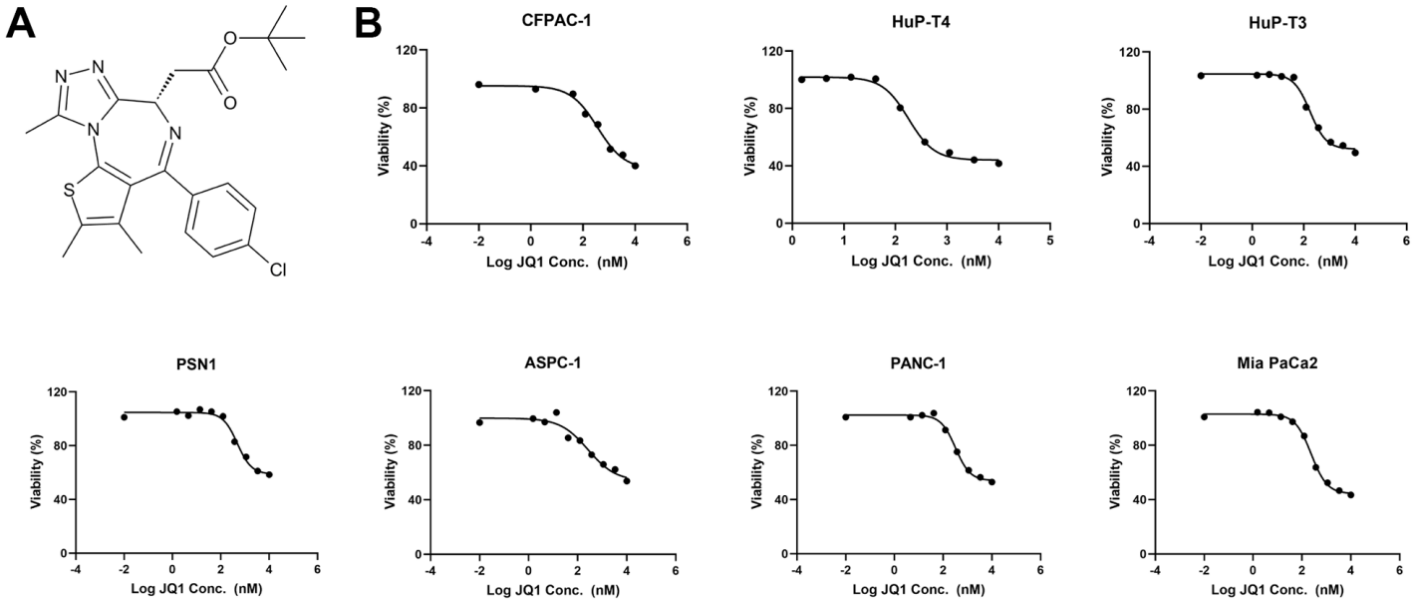
**The BET inhibitor JQ1 suppresses *KRAS* mutant pancreatic cancer cells.** (**A**) The structure of the BET inhibitor JQ1. (**B**) Fitting curve of cytostatic responses illustrated a decreasing concentration gradient of JQ1 treated with 7 *KRAS* mutant pancreatic cancer cell lines (AsPC-1, MIA PaCa-2, PANC-1, HuP-T4, HuP-T3, PSN1, and CFPAC-1).

To identify sensitivity or resistance to BET inhibitors, we demonstrated the structure of JQ1 (Figure 2A) and examined the antiproliferative activity of JQ1 in 7 PDAC cell lines. Cytostatic responses were also observed in all cell lines (Figure 2B). We observed that the IC50 values of AsPC-1, PANC-1, HuP-T3, and PSN1 were not reached even if the maximum concentration of 10 μM JQ1 was used (Supplementary Table 2). The IC50 values of the other three cell lines (HuP-T4, Mia PaCa-2, CFPAC-1) were 177.6 nM, 238.7 nM, and 362.3 nM, respectively.

### Synergistic effects elicited by combined trametinib and JQ1 treatment in pancreatic cancer

**Figure 3:**
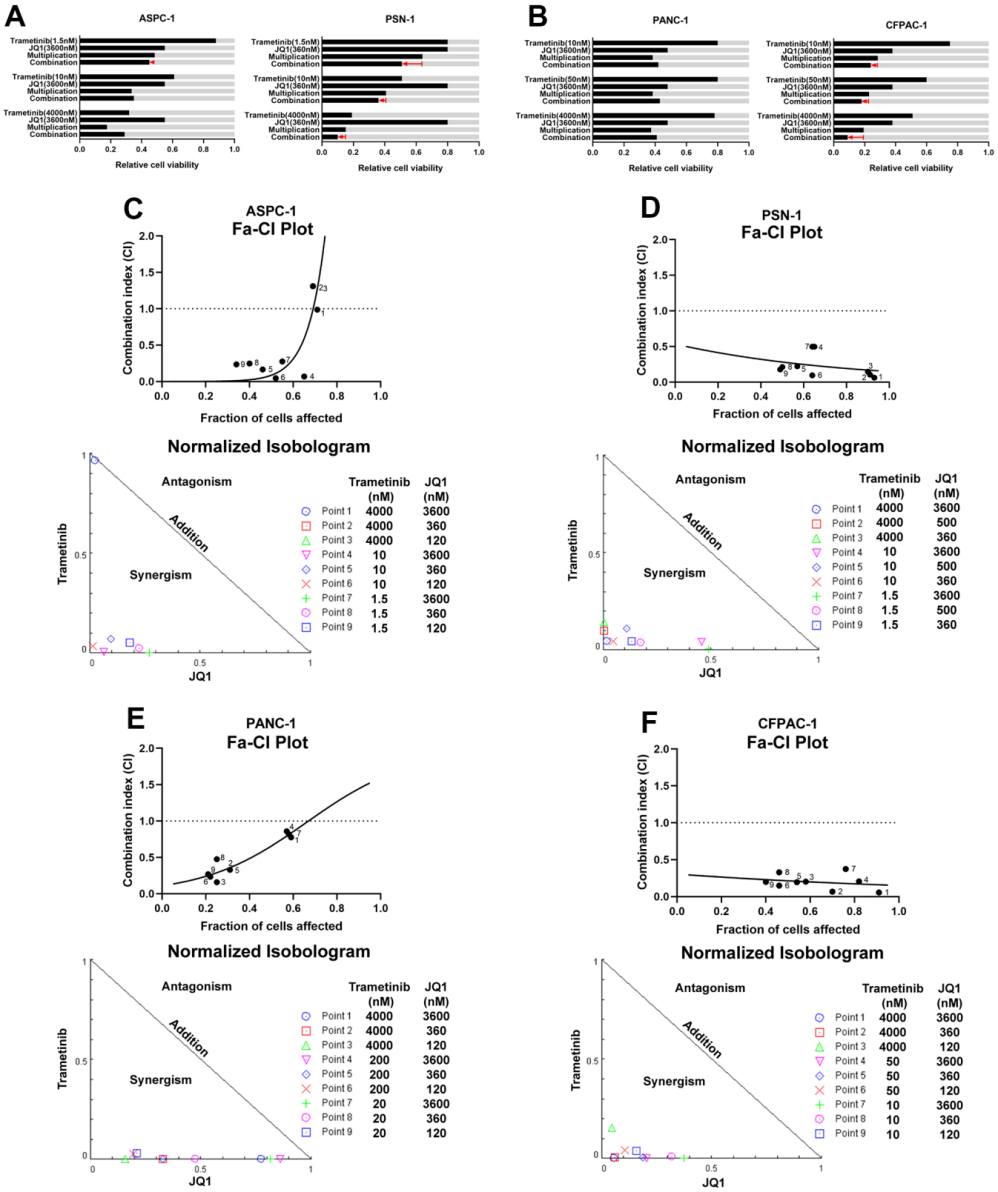
**Synergistic effects elicited by combined treatment trametinib and JQ1 in pancreatic cancer.** (**A**) Effect of trametinib and/or JQ1 on the percentage of cells in relative trametinib-sensitive PDAC cell lines (AsPC-1 and PSN-1). Light gray bars show control values. ‘‘Multiplication’’ indicates the expected effect of combined treatment if single-agent effects were multiplied; the red arrow indicates the actual effect of the combination. (**B**) Effect of trametinib and/or JQ1 on the percentage of cells in relative trametinib-resistant PDAC cell lines (PANC-1 and CFPAC-1). (**C**–**F**) Combination index (CI) (top) and isobologram (bottom) analyses reveal the synergistic effect of trametinib and JQ1 not only in trametinib-sensitive PDAC cell lines (AsPC-1 and PSN-1), but also in trametinib-resistant PDAC cell lines (PANC-1 and CFPAC-1). Fraction affected (Fa)-CI plots (top) and normalized isobolograms (bottom) are shown.

To confirm the inhibitory effect of blocking the RAS downstream pathway and BET epigenetic transcriptional pathway, we screened the activity of BET/MEK inhibitor combinations in human *KRAS* mutant PDAC cell lines. In trametinib-sensitive cell lines (AsPC-1 and PSN1), the combination of trametinib and JQ1 substantially reduced the percentage of cell viability, in AsPC-1 cells matching the multiplicative expectation and in PSN1 cells exceeding that which would be expected if monotherapy effects were multiplied (Figure 3A). In trametinib-resistant cells (CFPAC-1 and PANC-1), trametinib had little impact on cell viability. However, combined trametinib/JQ1 treatment resulted in a significantly greater reduction in cell viability than trametinib alone. In CFPAC-1 cells, the effect of trametinib and JQ1 combined was even stronger than would be expected if a single agent was used (Figure 3B). In PANC-1 cells, the effect of trametinib at a low concentration and JQ1 combination treatment still slightly exceeded expectations (Figure 3B). The following isobologram and combination index (CI) analyses demonstrated that combined trametinib/JQ1 treatment had synergistic inhibitory effects on both trametinib-sensitive and trametinib-resistant *KRAS* mutant PDAC cell growth for most concentration pairings (Figure 3C–3F). Except for AsPC-1 treated with a high concentration of trametinib, trametinib-sensitive cell lines with different combined treatment concentrations showed strongly synergistic inhibition with CI < 0.5 (Figure 3C, 3D). Interestingly, trametinib-resistant PDAC cell lines also displayed potent synergistic inhibitory effects of trametinib/JQ1 combination therapy (Figure 3E, 3F). It is worth mentioning that PANC-1 cells had almost no response to trametinib treatment alone, but there was a strong synergistic effect of combination therapy when JQ1 was used at low and median concentrations (Figure 3E). Together, the combined inhibition of the RAS downstream pathway and BET family proteins results in a potent synergistic antitumoral response to *KRAS* mutant pancreatic cancer cells.

### The combination of trametinib and JQ1 via different cell death modes inhibits pancreatic cancer

**Figure 4:**
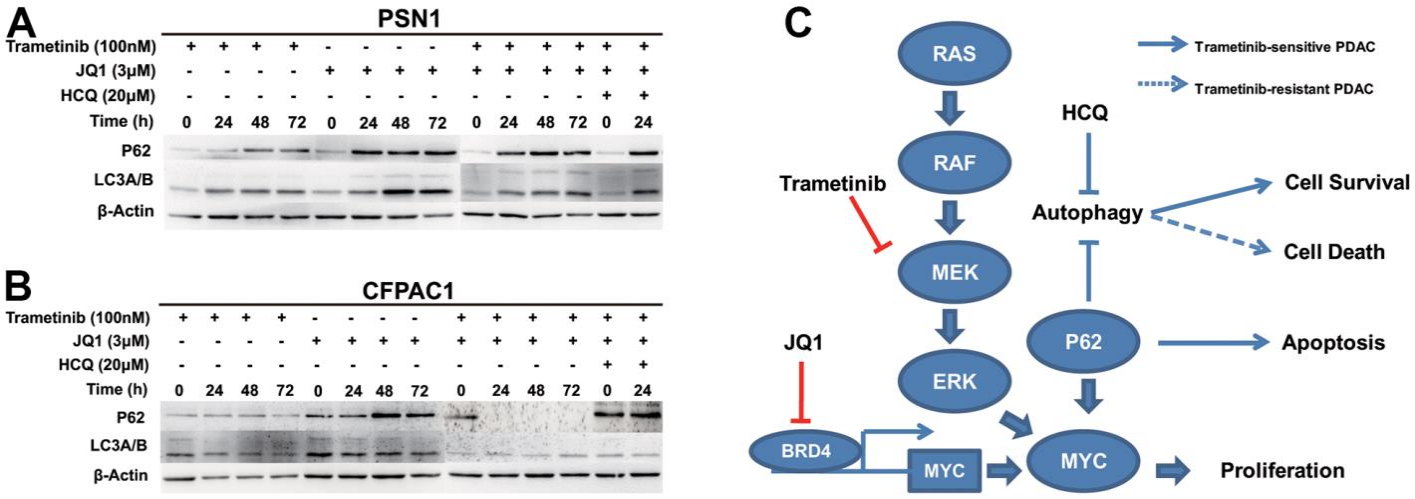
**The combination of trametinib and JQ1 via different cell death modes inhibits pancreatic cancer.** (**A**) Cell lysates prepared from PSN-1 cells treated with trametinib alone, JQ1 alone, trametinib+JQ1, and trametinib+JQ1+HCQ over a time course were analyzed by immunoblotting for p62, LC3, or actin, as indicated. (**B**) Cell lysates prepared from CFPAC-1 cells treated with trametinib alone, JQ1 alone, trametinib+JQ1, and trametinib+JQ1+HCQ over a time course were analyzed by immunoblotting for p62, LC3, or actin, as indicated. (**C**) Model of the synergistic effects induced by the combination treatment of trametinib and JQ1 in *KRAS* mutant pancreatic cancer.

Recently, it has been reported that inhibition of the RAS-MEK-ERK signaling pathway induces protective autophagy in pancreatic cancer cells preventing the cytotoxic effects of KRAS pathway inhibition [13]. Next, we preliminarily explored the mechanisms between autophagy and MEKi resistance and the synergistic effect with BET inhibitors. We examined the expression of autophagy-related proteins after treatment in the relative trametinib-sensitive cell line PSN-1 and the relative trametinib-resistant cell line CFPAC-1 by immunoblotting. We observed high expression of LC3 and accumulation of p62/SQSTM1 in PSN-1 cells treated with trametinib alone, JQ1 alone or the combination treatment, respectively (Figure 4A). Compared with trametinib alone, p62 expression was more increased in the combination treatment. It was suggested that the synergistic effect of PSN1 combined therapy inhibited autophagy, thus strengthening the apoptotic pathway. For the trametinib-resistant cell line CFPAC-1, the expression levels of LC3 and p62 did not change after treatment with trametinib alone (Figure 4B). Interestingly, the expression levels of LC3 were not altered in a time-dependent manner when combined with trametinib and JQ1 but were much lower than those of trametinib alone. However, p62 expression disappeared after combined treatment. The synergistic effect of CFPAC-1 combined therapy mainly activated autophagy-dependent cell death instead of apoptosis. To investigate whether the synergistic effect of the two different cell lines on combination therapy was involved in autophagy-dependent cell death, we added the autophagy inhibitor HCQ to PSN1 and CFPAC1 combination therapy. We found that the expression of p62 in PSN1 was slightly enhanced, while that in CFPAC1 was re-expressed after combined therapy plus HCQ (Figure 4A, 4B). This result indicated that autophagy-dependent cell death was mainly induced by the synergistic effect of combined therapy in trametinib-resistant cells but not in trametinib-sensitive cells.

## DISCUSSION

In this study, we screened the inhibitory effects of the MEK inhibitor trametinib and BET inhibitor JQ1 on 7 different pancreatic cancer *KRAS* mutant cell lines. Four cell lines that were relatively sensitive and resistant to trametinib were respectively tested for combination therapy. We observed a synergistic interaction from combination therapy on all cell lines, especially trametinib-resistant CFPAC-1 and trametinib-sensitive PSN1. Further mechanistic analysis showed that the combination therapy synergistic effect of trametinib-sensitive PDAC cells mainly came from apoptosis, while that of trametinib-resistant PDAC cells mainly activated autophagy-dependent cell death. This study was the first to clarify that combined trametinib and JQ1 treatment had a synergistic effect on *KRAS* mutant PDAC cells and elucidate that synergism induced different mechanisms of cell death in different PDAC cell lines.

Trametinib, an MEK1 and MEK2 kinase inhibitor, blocked ERK phosphorylation which downregulated MYC protein causing G1 cell cycle arrest and inducing apoptosis [14]. JQ1 a selective small-molecule bromodomain inhibitor, downregulated *MYC* transcription which produced a potent antiproliferative effect associated with cellular senescence and cell cycle arrest [9]. Combined treatment with BET and MEK inhibitors was reported to promote anaplastic thyroid tumors and colorectal cancer regression via synergistic suppression of *MYC* transcription [15, 16]. Recently, it has been demonstrated that combined MEK/BET inhibitors are much more effective depending on some biomarker in triple-negative breast cancer (TNBC) and in *KRAS* mutant non-small cell lung cancer (NSCLC) [17, 18]. However, the combinational effect of BET and MEK inhibitors has not been systematically evaluated in PDAC.

In our study, we found that either trametinib or JQ1 alone could inhibit the proliferation of some pancreatic cancer cell lines with *KRAS* alterations, irrespective of the mutational loci of *KRAS* and the mutational status of the other driver genes. Further studies demonstrated synergistic effects of the combination treatment of trametinib and JQ1 in both trametinib-resistant and trametinib-sensitive cell lines. It was shown that the BET inhibitor not only further enhanced the sensitivity of trametinib in trametinib-sensitive PDAC cells, but also improved the sensitivity of trametinib in trametinib-resistant PDAC cells. Finally, we preliminarily explored the mechanisms mediating the synergistic effects of the combination therapy in PDAC.

In the trametinib-sensitive PDAC cell line, the combined treatment definitely inhibited MYC, leading to an increase in p62 expression compared with trametinib alone, while LC3 expression at high levels changed little. It was elucidated that the synergistic effect of MEK/BET inhibitors mainly induced apoptosis in trametinib-sensitive cells, despite slight protective autophagy. In the trametinib-resistant PDAC cell line, the combination of MEK inhibitor and BET inhibitor dramatically decreased p62 expression compared with single drug, while p62 expression increased after anti-autophagy therapy was added. This result revealed that the synergistic effect of combination therapy mainly elicited autophagy-dependent cell death in trametinib-resistant cells. P62/SQSTM1, a ubiquitin-binding multifunctional protein linked to the extrinsic apoptosis pathway promoting programmed cell death, binds directly to LC3 family proteins to negatively regulate autophagy as a marker to study autophagic flux [19]. Autophagy is considered a mechanism by which cancer cells maintain high metabolic levels in poor nutritious environments [20]. Protective autophagy has generally emerged as a drug resistance mechanism inducing metabolic stress for cell survival when pancreatic cancer cells are treated with MEK or ERK inhibitors [13, 21]. However, under certain conditions such as anticancer treatment, autophagy can directly or indirectly induce cell death [20]. Our studies demonstrated that the synergistic effect of trametinib and JQ1 combined therapy might induce different ways of cancer cell death in different pancreatic cancer subtypes (Figure 3C). This indicates that the current clinical exploration of autophagy inhibitors combined with chemotherapy or trametinib in PDAC patients may encounter some bottlenecks [22]. We believe that only screening pancreatic cancer patients who produce protective or adaptive autophagy after treatment could obtain real benefits from anti-autophagic therapy.

Our study also has some limitations. We have not tested the synergistic effect of combination therapy *in vivo*. Animal assays to evaluate the safety and immune effect of trametinib/JQ1 combination therapy have been confirmed in other tumors [17, 18]. We preliminarily verified that blocking the *KRAS* downstream pathway combined with an anti-epigenetic BET inhibitor has a favorable synergistic effect in *KRAS* mutant PDAC cells. In addition, *KRAS* wild-type pancreatic cancer and the detailed regulatory molecular mechanism of different cell death modes induced by combined therapy should be explored and confirmed in the future. Finally, the mechanism of trametinib/JQ1/HCQ combined treatment is complex, and the antiproliferative effect and the cell death mode need strict designed experiments to be further evaluated.

In summary, our findings show that blocking RAS downstream signaling and epigenetic pathway synergistically increases the antiproliferative activity in *KRAS* mutant pancreatic cancer cells. Combination therapeutic synergism induces autophagy-dependent cell death in some pancreatic cancer subtypes. This suggests that trametinib and JQ1 can be viewed as potential combination therapeutic options for PDAC patients with *KRAS* alterations. Treatment containing anti-autophagic regimens requires screening suitable pancreatic cancer patients, which needs to be further verified.

## MATERIALS AND METHODS

### Cell culture

The human pancreatic cancer cell lines AsPC-1, MIA PaCa-2, PANC-1, HuP-T4, HuP-T3, PSN1, and CFPAC-1 were purchased from American Type Culture Collection (ATCC) and provided by Suzhou Truway Biotechnology Inc. All cell genetic information was analyzed and downloaded from the Cancer Cell Line Encyclopedia (CCLE) or Catalogu of Somatic Mutations in Cancer (COSMIC) database. Cells were cultured in RPMI 1640, McCoy's 5a, MEM or DMEM supplemented with 10% fetal bovine serum (FBS) and 1% penicillin/streptomycin under the recommended conditions. The ATCC has performed morphological, cytogenetic and DNA profile analyses for characterization of these cell lines. The cell passages were limited to 15 generations for all experiments in this study. Mycoplasma contamination was excluded using the antibiotic mycoplasmincin (InvivoGen) and was periodically examined using a MycoFluor Mycoplasma Detection Kit (Invitrogen, #M7006).

### Compounds

Trametinib (GSK1120212, MEK inhibitor, APExBio Technology, Shanghai, China), JQ1 (BET bromodomain inhibitor, APExBio Technology, Shanghai, China), and hydroxychloroquine (HCQ, autophagy inhibitor, APExBio Technology, Shanghai, China) were dissolved in DMSO.

### Cell viability assay

Cell viability assays were carried out using the CellTiter-Glo® Luminescent Cell Viability Assay (Promega, USA). Cells were seeded into 96-well cell culture plates at a density of 5000 cells per well in 100 μL of culture medium and treated with the indicated drugs at various concentrations. After 72 h of incubation, the cells were lysed with CellTiter Glo reagent (Promega, #G7573), and the luminescence signals produced by ATP molecules from live cells were measured using a SPARK microplate reader (TECAN, Switzerland) after 30 min of incubation at room temperature. The dose–response curve was fitted based on the relative survival cell percentage in nonlinear fitness (curve fit) using GraphPad Prism 8 software (http://www.graphpad.com/scientific-software/prism/). The software build-in analyses “nonlinear regression (curve fit)” and equation “log (inhibitor) vs. response-variable slope” were used for the data analysis and IC50 calculation.

### Immunoblot analysis

Cultured cells were extracted with RIPA buffer containing protease inhibitors and a phosphatase inhibitor cocktail (Roche). The protein concentration was determined by the BCA assay (Pierce). Proteins were resolved by SDS-PAGE, transferred to PVDF membranes (Millipore) and analyzed by immunoblotting. The antibodies used were as follows: LC3A/B antibody (#4108, 1:2000) and SQSTM1/p62 antibody (#5114, 1:2000) were purchased from Cell Signaling Technology, and β-actin (#A5316, 1:2500) was purchased from GenScript.

### Statistical analysis

All statistical analyses were performed using GraphPad Prism 8 (San Diego, CA, USA). Student’s unpaired t-tests were used to compare two independent groups before and after different treatments as appropriate. P values less than 0.05 were considered to be statistically significant.

## Supplementary Material

Supplementary Tables
