# Supplementary material for: Synergistic blocking of RAS downstream signaling and epigenetic pathway in KRAS mutant pancreatic cancer
Source: Aging (Albany NY). 2022 Apr 25;14(8):3597–606. doi: 10.18632/aging.204031 (PMC9085242; doi:10.18632/aging.204031)
Supplement: Supplementary Tables [file aging-14-204031-s001.pdf]

SUPPLEMENTARY TABLES

Supplementary Table 1. Parameters of proliferation curve fitting with trametinib in PDAC.

Cell lines	Bottom	Top	LogIC50	HillSlope	IC50(nM)
AsPC-1	26.22	122	0.01952	-0.3883	1.046
MIA PaCa-2	32.94	96.05	0.9622	-0.5241	9.167
PANC-1	42.92	104.9	3.013	-0.2029	1031
HuP-T4	54.26	194.6	-3.236	-0.2279	-
HuP-T3	61.18	102.2	3.335	-0.6284	-
PSN1	3.702	106	0.5873	-0.4583	3.866
CFPAC-1	45.41	107.8	1.787	-0.6881	61.22

Abbreviations: PDAC, pancreatic ductal adenocarcinoma.

Supplementary Table 2. Parameters of proliferation curve fitting with JQ1 in PDAC.

Cell lines	Bottom	Top	LogIC50	HillSlope	IC50(nM)
AsPC-1	53.65	99.85	2.423	-0.7698	-
MIA PaCa-2	44.3	102.9	2.378	-1.33	238.7
PANC-1	53.97	102.4	2.506	-1.448	-
HuP-T4	44.01	101.9	2.25	-1.738	177.6
HuP-T3	52.07	104.6	2.244	-1.375	-
PSN1	58.84	104.7	2.681	-1.478	-
CFPAC-1	37.7	95.25	2.559	-0.8447	362.3

Abbreviations: PDAC, pancreatic ductal adenocarcinoma.
